# Inhibitory Effect of *Sorbus aucuparia* Extracts on the *Fusarium proliferatum* and *F. culmorum* Growth and Mycotoxin Biosynthesis

**DOI:** 10.3390/molecules29174257

**Published:** 2024-09-08

**Authors:** Sylwia Ryszczyńska, Natalia Gumulak-Wołoszyn, Monika Urbaniak, Łukasz Stępień, Marcin Bryła, Magdalena Twarużek, Agnieszka Waśkiewicz

**Affiliations:** 1 Department of Chemistry, Faculty of Forestry and Wood Technology, Poznań University of Life Sciences, Wojska Polskiego 75, 60-625 Poznań, Poland; sylwia.ryszczynska@up.poznan.pl; 2 Department of Forest Ecosystem Protection, Faculty of Forestry, University of Agriculture in Kraków, Aleja 29 Listopada 46, 31-425 Kraków, Poland; natalia.gumulak@student.urk.edu.pl; 3 Plant-Pathogen Interaction Team, Institute of Plant Genetics, Polish Academy of Sciences, Strzeszyńska 34, 60-479 Poznań, Poland; murb@igr.poznan.pl (M.U.); lste@igr.poznan.pl (Ł.S.); 4 Department of Food Safety and Chemical Analysis, Prof. Waclaw Dabrowski Institute of Agricultural and Food Biotechnology—State Research Institute, Rakowiecka 36, 02-532 Warsaw, Poland; marcin.bryla@ibprs.pl; 5 Department of Physiology and Toxicology, Faculty of Biological Sciences, Kazimierz Wielki University, Chodkiewicza 30, 85-064 Bydgoszcz, Poland; twarmag@ukw.edu.pl

**Keywords:** *Sorbus aucuparia*, *Fusarium* spp., ergosterol, mycotoxins, supercritical fluid extraction, chromatographic analysis

## Abstract

Fungal infections are among the most common diseases of crop plants. Various species of the *Fusarium* spp. are naturally prevalent and globally cause the qualitative and quantitative losses of farming commodities, mainly cereals, fruits, and vegetables. In addition, *Fusarium* spp. can synthesize toxic secondary metabolites—mycotoxins under high temperature and humidity conditions. Among the strategies against *Fusarium* spp. incidence and mycotoxins biosynthesis, the application of biological control, specifically natural plant extracts, has proved to be one of the solutions as an alternative to chemical treatments. Notably, rowanberries taken from *Sorbus aucuparia* are a rich source of phytochemicals, such as vitamins, carotenoids, flavonoids, and phenolic acids, as well as minerals, including iron, potassium, and magnesium, making them promising candidates for biological control strategies. The study aimed to investigate the effect of rowanberry extracts obtained by supercritical fluid extraction (SFE) under different conditions on the growth of *Fusarium* (*F. culmorum* and *F. proliferatum*) and mycotoxin biosynthesis. The results showed that various extracts had different effects on *Fusarium* growth as well as ergosterol content and mycotoxin biosynthesis. These findings suggest that rowanberry extracts obtained by the SFE method could be a natural alternative to synthetic fungicides for eradicating *Fusarium* pathogens in crops, particularly cereal grains. However, more research is necessary to evaluate their efficacy against other *Fusarium* species and in vivo applications.

## 1. Introduction

*Sorbus aucuparia* is a deciduous tree species native to most of Europe and parts of Asia and Africa, commonly cultivated as an ornamental plant, which has a few climate requirements, as it can occur in both high mountains with low temperatures and the hot south. It is a species whose fruits are edible but neglected because of their natural bitter taste and the necessity of processing them before consumption to make them palatable. In some regions, particularly in Eastern Europe and Scandinavia, it is used as an ingredient in food products such as jams or syrups [1]. Rowan is rich in organic acids, including ascorbic acid, and phenolic compounds, which have shown a wide range of biological properties [2,3]. Substances extracted from rowan fruits have proven to exhibit diuretic, anti-inflammatory, vasoconstrictor, and anti-diabetic properties [4]. The rowan tree is an important biocenotic species that provides food for many birds, e.g., song thrush (*Turdus philomelos*), Eurasian bullfinch (*Pyrrhula pyrrhula*), and mistle thrush (*Turdus viscivorus*) [5]. It is also a food ingredient for mammals, such as the European pine marten (*Martes martes*) [6], roe deer (*Capreolus capreolus*) [7], and red foxes (*Vulpes vulpes*) [8], establishing the importance of mammals as dispersers of rowan seeds [8]. Due to the constant rowanberry seed multiplication, high variability is observed for most morphological and biochemical traits of wild fruits. Therefore, it is important to learn about the characteristics of specific individuals and to introduce breeding for valuable fruits [9].

Supercritical fluid extraction is an advanced extraction method utilizing supercritical fluids, typically carbon dioxide (CO_2_), as a solvent. During the SFE process, the supercritical CO_2_ is passed through the material containing the desired compounds. The solvating properties of supercritical CO_2_ can be tuned by adjusting pressure and temperature, allowing selective extraction of specific compounds, which enables precise targeting of desired substances while leaving unwanted components behind. Compared to traditional solvent extraction, SFE operates at lower temperatures, preserving sensitive compounds, and uses non-toxic CO_2_, making it environmentally friendly and leaving no solvent residues [10]. Using SFE for *Sorbus aucuparia* fruits holds promise for obtaining bioactive compounds, allowing for selective extraction of beneficial components while maintaining the fruit’s nutritional integrity. The resulting extract may be a rich source of bioactive compounds for food, pharmaceutical, and biotechnological industries. Moreover, due to the variety of natural compounds, rowan fruit extracts prepared by the SFE method may have antimicrobial properties, which can be used to inhibit the growth of mycopathogens.

Up to now, a few articles have been published on the antimicrobial properties of *Sorbus aucuparia* fruit extracts. The antimicrobial effect is mainly attributed to the high content of sorbic acid and biphenyl phytoalexins, particularly aucuparin in rowanberries [11,12,13]. Most of the previous research concerns the effect of extracts obtained from *Sorbus aucuparia* fruits on bacteria growth [14,15]. In the research of Liepiņa et al. (2013), the highest inhibition on *Bacillus cereus* had ethanolic and aqueous extracts obtained from fresh rowanberries, which reached a percentage of inhibition equal to 10% and 9.7%, respectively [16]. A very strong antibacterial effect of acetone-derived phenolic-rich fractions of rowanberry extract against *B. cereus* was found [17], as well as for methanolic and aqueous extracts, which achieved an inhibition percentage above 75% [18]. Antibacterial properties of *Sorbus aucuparia* fruit extracts have also been observed for *Staphylococcus aureus*, *Escherichia coli*, *Pseudomonas aeruginosa*, *Salmonella enterica*, *Bacillus subtilis*, *Serratia marcescens*, and *Campylobacter jejuni* [16,17,18]. Regarding the antifungal activity of *Sorbus* extracts, only a few studies have been conducted, mainly for *Candida albicans*, and their results are inconclusive [16,17,19,20,21,22,23].

Fungal infections stand as some of the most destructive agricultural diseases worldwide. Within the *Fusarium* spp., *F. culmorum* and *F. proliferatum* are widespread, especially in Europe, and cause substantial losses in cereals, cereal-based items, fruits, and vegetables [24,25,26]. Crop yield reduction caused by fusariosis reached from 10 to 40% [27]. These *Fusarium* species synthesize mycotoxins under favorable conditions, which are high temperature, humidity, and moisture, occurring pre-harvest, post-harvest, during processing, or even in storage [28]. Additionally, most mycotoxins produced by *Fusarium* spp. exhibit heat stability and raise serious health concerns in humans and farm animals, leading to mutagenic, teratogenic, neurotoxic, and carcinogenic effects [29]. *Fusarium* spp. are associated with diseases in people with localized or invasive infections who have a history of immunosuppression [30]. These infections are difficult to treat mainly due to drug-resistant isolates [31]. Due to their characteristics, *Fusarium* fungi become resistant to fungicide exposure very quickly [32]. The crucial thing, therefore, is the implementation of appropriate methods or fungicides [33].

This work aims to investigate the antifungal properties of *Sorbus aucuparia* fruit extracts obtained by supercritical fluid extraction under various temperature and pressure conditions. The antifungal activity of rowanberry fruit extracts was assessed against two *Fusarium* strains: *F. proliferatum* and *F. culmorum*. The effect of the extract treatments on mycelial growth and mycotoxin biosynthesis was assessed by qualitative and quantitative chromatographic analysis of ergosterol and secondary fungal metabolites.

## 2. Results

### 2.1. Preparation of the Sorbus aucuparia Fruit Extracts by Supercritical Fluid Extraction Method

Rowanberry extracts were obtained by the SFE method according to the further described procedure in four variants differing in extraction parameters such as temperature and pressure (Table 1).

The extraction yield ranged from 16.93 to 18.05%. The most efficient extraction conditions were demonstrated at 70 °C and 300 bar (variant E3). It has also been shown that higher pressure in the extraction process had a significant impact on the increase in extraction yield (extracts E3 or E1, *p* < 0.05), while lower pressure, regardless of the temperature value, resulted in a similar extraction yield.

### 2.2. Total Phenolic Content of the Methanolic and Aqueous Extracts

**Figure 1:**
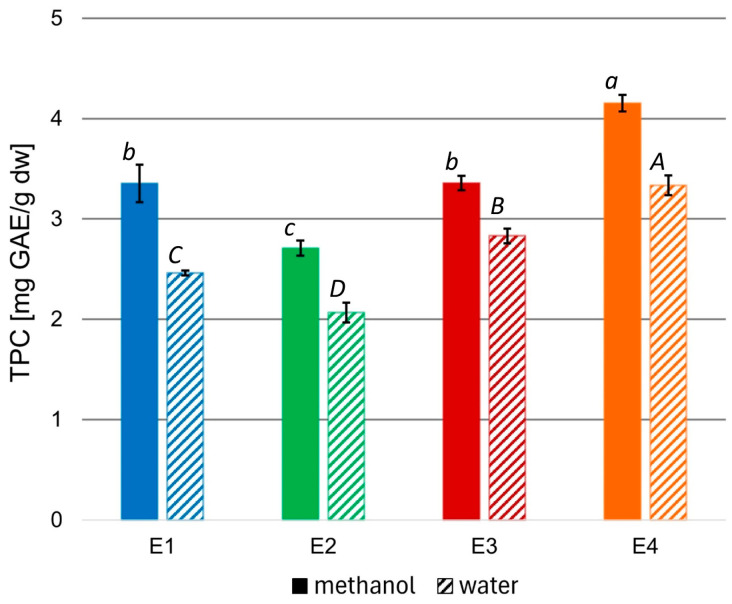
TPC in methanolic and aqueous rowanberry extract variants E1–E4. All values are means of three replicates; error bars represent standard deviation. The values assigned with the superscripts of different letters are significantly different (Tukey’s HSD test, significant at *p* < 0.05).

To evaluate the bioactivity of the prepared extracts, the total phenolic content (TPC) of the methanolic variants, as well as the aqueous variants obtained from the methanolic ones, were measured, and the results were expressed as mg of gallic acid (GAE) per 1 g of extract dw (Figure 1).

The TPC of the rowanberry methanolic extracts ranged from 2.71 to 4.15 mg GAE/g dw. However, aqueous variants had significantly lower TPC than corresponding methanolic ones (*p* < 0.05), reaching values from 2.07 to 3.33 mg GAE/g dw. The highest TPC values characterized the extract variant E4, regardless of the solvent, while the lowest—extract variant E2.

### 2.3. The Inhibitory Effect of Sorbus aucuparia Fruit Extracts on Fusarium spp. Growth

**Figure 2:**
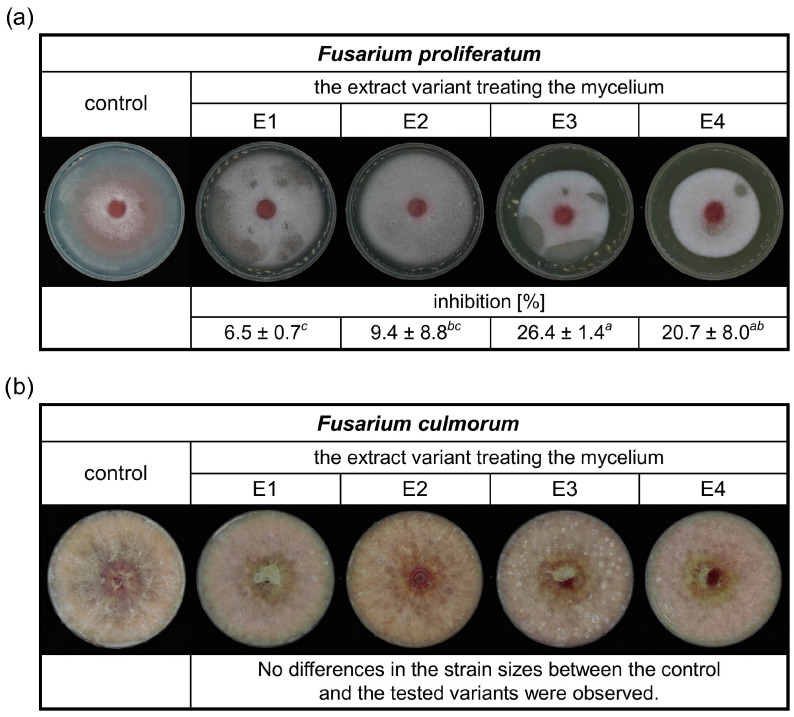
The inhibitory effects of different rowanberry extracts (E1–E4) on *F. proliferatum* (**a**) and *F. culmorum* (**b**) mycelia growth in the medium. All values are means of three replicates ± standard deviation. The values assigned with the superscripts of different letters are significantly different (Tukey’s HSD test, significant at *p* < 0.05).

This study focused on the antifungal activity of rowanberry extracts obtained by the SFE against *Fusarium* spp. Therefore, the effect of the obtained aqueous extract variants (E1–E4) on the growth of *F. proliferatum* and *F. culmorum* was evaluated, and the results are presented in Figure 2.

It was observed that the prepared extracts had an inhibitory effect on the growth of *F. proliferatum* (Figure 2a). The lowest inhibition of the extracts was 6.5%. The most significant antifungal activity against *F. proliferatum* was exerted by the extract variant E3, with a reduction in mycelial growth of 26.4%. In the case of the inhibition study of rowanberry extracts on the *F. culmorum* growth, no differences between the control and the tested variants were observed (Figure 2b).

### 2.4. The Effect of Sorbus aucuparia Fruit Extracts on Ergosterol Content

**Figure 3:**
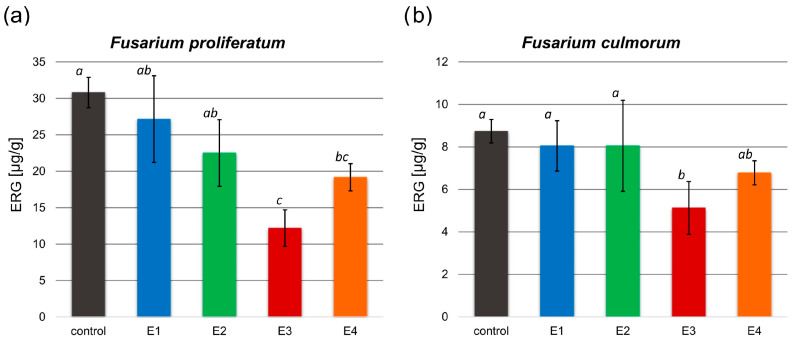
Ergosterol (ERG) content [µg/g] in the control and tested samples treated with the obtained extracts (E1–E4) after inoculation with *F. proliferatum* (**a**) and *F. culmorum* (**b**). All values are means of three replicates; error bars represent standard deviation. The values assigned with the superscripts of different letters are significantly different (Tukey’s HSD test, significant at *p* < 0.05).

The antifungal activity of the obtained extracts on the *F. proliferatum* and *F. culmorum* growth was investigated based on the analysis of the ergosterol (ERG) content in the control and tested samples (Figure 3).

In both examined *Fusarium* spp. treated with the rowanberry extracts, ERG content decreased compared to the control group. A significant difference in ERG concentration was observed after treating *F. proliferatum* with each of the obtained extract variants, but only the E3 and E4 extract variants had a significant effect on *F. culmorum* (*p* < 0.05). The ERG content of the tested samples was in the range between 12.18 and 27.15 µg/g (30.79 µg/g control) for *F. proliferatum* (Figure 3a) and 5.13–8.04 µg/g (8.73 µg/g control) for *F. culmorum* (Figure 3b). The most significant decrease in ergosterol concentration (*p* < 0.05) was observed in samples treated with the extract variant E3.

The ERG reduction (Table 2) in samples with the addition of *Sorbus aucuparia* fruit extracts ranged from 12.41 to 60.67% for *F. proliferatum* and from 7.70 to 41.62% for *F. culmorum*, depending on the extraction variants. The greatest reduction in ergosterol content compared to the control group was observed after treatment of both *Fusarium* spp. with extract variant E3.

### 2.5. Mycotoxin Identification

**Figure 4:**
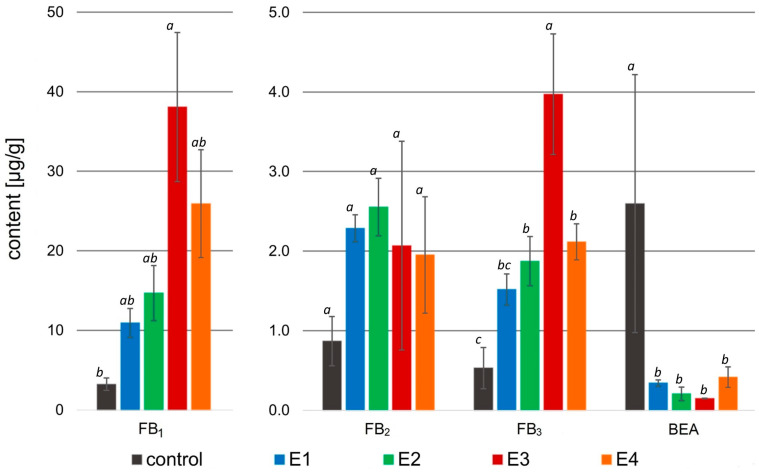
Mycotoxin content [µg/g] in the samples treated with rowanberry extracts (four variants, E1–E4) after inoculation with *Fusarium proliferatum*. FB_1_—fumonisin B_1_, FB_2_—fumonisin B_2_, FB_3_—fumonisin B_3_, BEA—beauvericin. All values are means of three replicates; error bars represent standard deviation. The values assigned with the superscripts of different letters are significantly different (Tukey’s HSD test, significant at *p* < 0.05).

In this study, the multi-mycotoxins method was used to analyze the effect of rowanberry extracts on mycotoxin biosynthesis by *Fusarium* spp. For *F. proliferatum*, the content of fumonisins—B_1_ (FB_1_), B_2_ (FB_2_), and B_3_ (FB_3_), and beauvericin (BEA) were determined. However, in the case of *F. culmorum*, among the 12 analyzed mycotoxins—deoxynivalenol (DON), 3- and 15-acetyldeoxynivalenol (3- and 15-AcDON), deoxynivalenol-3-glucoside (DON-3G), nivalenol (NIV), nivalenol-3-glucoside (NIV-3G), fusarenon X (FUSX), zearalenone (ZEN), zearalenone-14-sulfate (ZEN-14S), zearalenone-14-glucoside (ZEN-14G), α-zearalenol (α-ZOL), and β-zearalenol (β-ZOL)—only six were identified and quantified: DON, 3- and 15-AcDON, ZEN, ZEN-14S, and α-ZOL (Figure 4).

The application of rowanberry extracts to *Fusarium proliferatum* resulted in an intriguing effect on mycotoxin biosynthesis. A significant decrease in BEA content in the samples treated with the rowanberry extracts was observed, ranging from 0.15 to 0.42 to μg/g compared to the control group—2.60 μg/g (*p* < 0.05). However, fumonisins were biosynthesized by *F. proliferatum* more prominently in the samples exposed to the prepared extracts. The highest concentration of FB_1_ (38.08 μg/g) and FB_3_ (3.97 μg/g) in the prepared samples was connected with the addition of extract variant E3 (control: 3.23 μg/g, and 0.53 μg/g, respectively). However, the highest content of FB_2_ (2.55 μg/g) was observed after the addition of the extract variant E2 (control: 0.87 μg/g), whereas the differences in the production of this fumonisin by each extract were not statistically significant (*p* > 0.05). The total content of the fumonisins in the tested samples with *F. proliferatum* treated with the rowanberry extracts was: 14.72, 19.09, 44.11, and 29.98 µg/g for E1, E2, E3, and E4 variants, respectively (control: 4.63 µg/g). The most significant difference in the content of fumonisins was observed between the control sample and the sample treated with the E3 extract variant (*p* < 0.05).

In the case of the *Fusarium proliferatum*, the percentage of BEA reduction by prepared fruit extracts reached the highest value of 92.1% after treatment with variant E3, whereas this result is statistically similar to other extract variants (*p* > 0.05, Table 3). As previously mentioned, no reduction was observed in the content of fumonisins—in fact, their biosynthesis exhibited an increase compared to the control group. In the case of variant E3, a significant increase in the production of FB_1_ (1017.9%) and FB_3_ (716.1%) was observed (*p* < 0.05). However, the increase in FB_2_ production was at a similar level (123.1–208.5%), regardless of the used extract variant.

**Figure 5:**
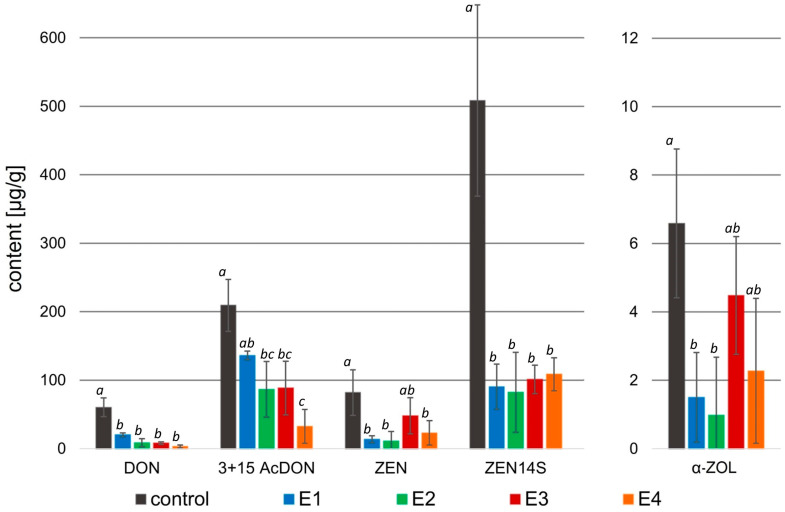
Mycotoxin content [µg/g] in the samples treated with rowanberry extracts (four variants, E1–E4) after inoculation with *Fusarium culmorum*. DON—deoxynivalenol, 3 + 15 AcDON—3- and 15-acetyldeoxynivalenol, ZEN—zearalenone, ZEN14S—zearalenone-14-sulfate, α-ZOL—α-zearalenol. All values are means of three replicates; error bars represent standard deviation. The values assigned with the superscripts of different letters are significantly different (Tukey’s HSD test, significant at *p* < 0.05).

Instead, the biosynthesis of all mycotoxins by *F. culmorum* after treatment with the rowanberry extracts was significantly lower compared to the control group (*p* < 0.05 for all determined mycotoxins; Figure 5).

In the case of *F. culmorum*, the addition of all extract variants resulted in a significant decrease in the DON (3.14–20.21 μg/g), as well as 3- and 15-AcDON (32.45–136.12 μg/g) content compared to the control group (60.30 and 209.36 μg/g, respectively). The greatest decrease in the concentration of these mycotoxins was observed after the treatment of *F. culmorum* with the extract variant E4. However, in the case of DON, the differences in the effects of individual extracts were not significant (*p* > 0.05), while a significant difference was observed in the case of 3- and 15-AcDON content after treatment with E4 (*p* < 0.05). Moreover, all extract variants decreased in ZEN (11.10–48.01 μg/g), ZEN-14S (82.32–108.61 μg/g), and α-ZOL (0.98–4.48 μg/g) concentrations in comparison to the control (81.75, 508.53, and 6.58 μg/g, respectively). The highest decrease in these mycotoxin contents was caused by the addition of the extract variant E2 to the mycelium, whereas in the case of ZEN and α-ZOL, there were no significant differences between the activity of extract variants E1 and E2 (*p* > 0.05), but for the concentration of ZEN-14S, a significant difference caused by the extract variant E2 was observed (*p* < 0.05).

The application of rowanberry extracts on *F. culmorum* resulted in a reduction in all detected mycotoxins by at least 32.7%, whereas the different extract variants inhibited mycotoxin production in various grades (Table 4). A similar reduction in mycotoxins (*p* < 0.05) was observed regardless of the extract variant for DON: 86.6–95.1% (exception: E1: 66.1%), ZEN: 74.7–89.1% (exception E3: 43.6%), and ZEN-14S: 78.7–82.5%. The extract variant E4 reduced the synthesis of 3- and 15-AcDON by *F. culmorum* the most (85.5%), but the extract variant E2 reduced the synthesis of α-ZOL by 89.2% (*p* < 0.05).

## 3. Discussion

Despite the considerable advancements in modern agriculture, fungal infections affecting agricultural produce, notably fusariosis, persist as a substantial threat [34]. Consequently, there is a critical need to develop innovative methodologies against pathogenic fungi. A promising approach involves utilizing biological methods to inhibit fungal development in crops, e.g., through the use of plant extracts possessing antimicrobial properties [35]. One such natural candidate is rowanberry, known for its richness in organic acids as well as phenolic and flavonoid compounds [14]. However, the imperative involves the judicious selection of an appropriate extraction method and conditions to obtain the most effective extract.

Supercritical fluid extraction is an innovative and promising method offering significant advantages in the extraction of natural compounds while aligning with the growing demand for environmentally sustainable and high-quality extraction [36]. This approach is currently widely used for the extraction of essential oils from, e.g., *Lavendula genus*, *Pimpinella anisum*, or *Jasminum sambac* [37,38,39]. In the case of *Sorbus aucuparia*, the SFE technique was used to extract pomace, seeds, or waste to obtain oil extracts [40,41,42]. The highest yield of the lipophilic fraction was received by Bobinaite et al. (2020) at 60 °C and 450 bar in 180 min [40]. However, Ivakhnov et al. (2019) reached the highest yield of the oil fraction, equal to 9.02 wt % (weight percent) at 85 °C and 329.3 bar in 72 min [42]. Testing modified extraction (CO_2_ expanded ethanol combined with sonication) with different co-solvents for receiving lipids from berry seeds, including rowanberries, showed that the optimal operating conditions were as follows: ethanol, 52 °C, 100 bar, and 7 min [41]. However, no research has been carried out on other (aqueous) fractions of extracts obtained, especially from *Sorbus aucuparia* fruits. Due to divergent data on extraction parameters, we decided to prepare four different extract variants using low and high temperatures (40 and 70 °C) at intermediate pressure values (200 and 300 bar). We used methanol as a co-solvent, which is a commonly used solvent in numerous extractions. To prepare the richest extracts, the extraction time was set to 180 min. The highest extraction yield was obtained when high temperature and pressure were used (extract variant E3), while the lowest yield was caused by the use of 200 bar, without a significant influence of temperature (E2 and E4). The extraction yield ranged from 16.90 to 18.05% (169.60–184.40 g/kg) and was similar to the yields of the typical extractions of rowanberry pomace using methanol, e.g., 20.75% [43]. In general, at a specific temperature, an increase in pressure increases the density of the solvent and the solubility of targeted compounds, promoting an increase in extraction yield. Under constant pressure, the temperature increase decreases the solvent density, reducing its solvating power but improving the vapor pressure, which increases the analyte solubility, thus increasing extraction efficiency [44]. The obtained results are consistent with these data.

To assess the quality of the obtained rowanberry extracts, the total phenolic contents were measured (see Figure 1). To date, the highest TPC values of the defatted methanol–water rowanberry fruit extract (1:1, *v*/*v*) were equal to 26.03 mg GAE/g dw and of the water residue—14.27 mg GAE/g dw [45]. However, most reports on methanolic extracts of *Sorbus aucuparia* fruits reported lower TPC values in a range of 2.53–10.05 mg GAE/g, depending on rowanberries genotypes, 4.35–8.19 mg GAE/g for different cultivars, and between 2.68 and 3.35 mg GAE/g [13,46,47,48]. The extract variant E4 prepared at 70 °C and 200 bar was characterized by the highest TPC (4.15 and 3.33 mg GAE/g dw for methanol and water, respectively). In comparison, the extract variant E2 obtained at 40 °C and the same pressure had the lowest TPC (2.71 and 2.07 mg GAE/g dw for methanol and water, respectively). These results suggest that higher temperatures may promote the release of phenolic compounds from the tested material due to the improvement of their solubility or acceleration of diffusion connected with the increasing temperature [49]. Aqueous solutions prepared from the corresponding methanolic extracts had significantly lower TPC values, which can be explained based on the different solubility of phenolic compounds in water and methanol. Nevertheless, the content of phenolic compounds in aqueous extracts was substantial, and due to their potential to provide a biocompatible environment given their applications as fungicides and ensuring the safety of both plants and their consumers, we chose them for microbiological experiments.

Previously published research on rowan fruit extracts has primarily concentrated on their antibacterial properties, with limited studies on their antifungal effects, which have yielded inconclusive results. No inhibitory effect on *Candida albicans* growth was observed after treatment with the aqueous and ethanolic, as well as acetone-derived phenolic-rich, rowanberry extracts [16,17]. However, Maliuvanchuk et al. (2023) determined an inhibition zone of ethanolic fruit extract obtained by the percolation method on *C. albicans* equal to 5.81 mm [23]. Further, an antifungal effect was not observed against *Rhizopus stolonifer* treated with ethanolic–aqueous rowanberry extract [19]. Importantly, several reports present promising results regarding the growth inhibition of various fungal species by extracts obtained from the other *Sorbus* spp. The extracts prepared from *S. sibirica* fruits were able to inhibit the growth of *Aspergillus niger* (fungistatic diameter: 1.51 mm) [50]. Moreover, antifungal properties of *S. cashmiriana* whole plant extracts were tested on various fungal species, and the most efficient inhibition was obtained against *Aspergillus flavus* (68%) and the lowest against *Rhizoctonia solani* (24%) [20]. However, acidified ethanolic extracts obtained from *S. domestica* fruits did not show antifungal activity against *C. albicans* and *Saccharomyces cerevisiae* [21]. In our experiment, the addition of rowanberry extracts inhibited the growth of *Fusarium proliferatum* from 6.5 to 26.4%, but for *F. culmorum*, growth inhibition was not observed (see Figure 2). However, to determine the actual antifungal activity of the obtained extracts, we measured ergosterol content in the treated mycelia. ERG, which is a sterol compound found in the cell membranes of fungi that serves as a crucial component for maintaining the structural integrity and fluidity of fungal cell membranes, is used as a specific biomarker to quantify fungal biomass [22]. A reduction in ERG content was observed for all samples, but extract variant E3 was the most effective (ERG reduction: 60.67% for *F. proliferatum* and 41.62% for *F. culmorum*). It is noteworthy that the extraction yield of this particular variant was the highest, potentially indicating an increased concentration of bioactive compounds, which exhibit antifungal properties, but this is not directly related to the total content of phenolic compounds. The extracts obtained at low temperature (40 °C) had significantly lower antifungal properties, but still, their decrease in ERG content was not lower than 7.70% against *F. culmorum* and 12.41% against *F. proliferatum*. In the case of *F. proliferatum*, ERG concentration reduction corresponded to the observed inhibition of mycelium growth. However, the reduction in ERG content with the absence of differences in strain sizes between the control and the tested samples of *F. culmorum* may be caused by the specific structure of the mycelium, for which it was not possible to observe a size decrease.

The promising effects of the microbiological studies induced us to analyze secondary metabolites of *Fusarium* spp. *F. proliferatum* produced beauvericin and fumonisins from the B group. All tested rowanberry extracts significantly decreased the production of BEA, reaching reduction values from 80.6 to 92.1% (without significant differences in the degree of reduction between individual extracts, *p* > 0.05). Interestingly, in the case of fumonisins, there was an enormous increase in their biosynthesis in all samples after extract addition (see Figure 4). So far, this effect has been only described in a few reports [51,52]. This phenomenon can be interpreted as part of a stress response mechanism caused by the external factor, where the fungi change metabolic priorities [53]. Under stress caused by the presence of rowanberry extracts, *F. proliferatum* reallocates resources to prioritize the synthesis of compounds that enhance their survival, like fumonisins, which disrupt sphingolipid metabolism in host organisms [54]. Moreover, the significant increase in fumonisin production may divert metabolic resources away from beauvericin biosynthesis, or the regulatory pathways controlling BEA synthesis might be directly inhibited by the stress, further reducing its production. Furthermore, under stress conditions, fungi can release alkaline proteases, which, further, are able to break peptide bonds presented in the BEA structure [55]. Instead, *Fusarium culmorum* synthesized B-trichothecenes (DON, 3- and 15-AcDON) and zearalenone with derivatives (ZEN, ZEN-14S, α-ZOL). The simultaneous presence of free and modified forms can be attributed to the intricate interplay between the fungus and its host environment. Modified mycotoxins may originate from the metabolic pathways of infected plants or be synthesized de novo by the fungus, leading to their coexistence alongside their free forms [56]. The obtained results showed that *F. culmorum* produced ZEN-14S in high concentration (control: 508.53 μg/g) and α-ZOL in low concentration (control: 6.58 μg/g). The application of rowanberry fruit extracts resulted in a notable decrease (*p* < 0.05) in the biosynthesis of all tested mycotoxins, with reductions not less than 32.7%.

## 4. Materials and Methods

### 4.1. Plant Material

The plant material consisted of ripe fruits of the rowan tree *Sorbus aucuparia* obtained from trees growing in Poznań and Zagórów (Central Poland). The fruits were collected directly from the tree, then lyophilized and ground in liquid nitrogen. The material was stored frozen at −20 °C.

### 4.2. Fungal Material

*Fusarium proliferatum* (PEA 1) and *Fusarium culmorum* (KF 846) were isolated from pea seeds and wheat kernels, respectively. They were identified by molecular techniques [35,57] and preserved at the Institute of Plant Genetics, Polish Academy of Science, Poznań, Poland.

### 4.3. Reagents

Carbon dioxide CO_2_ (99.9995%, Air Products, Warszawa, Poland), methanol (99.5%, Chempur, Piekary Śląskie, Poland), and deionized water (Milipore, Burlington, MA, USA) were used to prepare rowanberry extracts. Potato dextrose agar (PDA) used for the in vitro experiment was supplied by Oxoid, Basingstoke, UK. Sodium hydroxide (≥98.8%, POCH, Gliwice, Poland), hydrochloric acid (≥37%, Sigma Aldrich, Taufkirchen, Germany), methanol (99.99%, POCH, Gliwice, Poland), and pentane (99%, POCH, Gliwice, Poland) were used to extract ergosterol or mycotoxins. For the HPLC measurements, the appropriate solvents with LC grade were used. Analytical standards purchased as ready-to-use solutions from Romer Labs (Tulln, Austria) included ERG, FB_1_, FB_2_, FB_3_, BEA, DON, DON-3G, 3-AcDON, 15-AcDON, NIV, NIV-3G, FUSX, ZEN, and ZEN-14G (100 µg/mL). The α- and β-ZOL concentrations were 10 µg/mL. ZEN-14S (100 µg/mL) was purchased from Aokin (Berlin, Germany).

### 4.4. Supercritical Fluid Extraction

The extraction was performed utilizing the MV-10ASFE extractor (Waters, Manchester, MA, USA), which included a CO_2_ cylinder, cooling system, fluid delivery module, column oven, back pressure regulator, heat exchanger, and fraction collection module, as well as ChromScope v1.20 software (Waters, Manchester, MA, USA). The extraction of 25 g of rowanberries conducted under various temperature (40 and 70 °C) and pressure (200 and 300 bar) conditions enabled the preparation of 4 different extract variants (E1–E4). The CO_2_ flow rate was 4 mL/min, and the methanol (co-solvent) flow was 1 mL/min. Each experimental run took 180 min, with the first dynamic time of 15 min, the static time of 30 min, and the second dynamic time of 135 min. The extraction yield was calculated using the following formula:

(1) $$extraction\;yield=\frac{m_{a-}m_{b}}{m_{a}}\times 100\%$$

where *m_a_* is the mass of *Sorbus aucuparia* fruits taken for extraction, and *m_b_* is the mass of the remaining rowanberry residues after extraction.

The initial methanolic extracts obtained from *Sorbus aucuparia* fruits were transferred to deionized water, the remaining methanol was removed, and the aqueous extracts were concentrated using an evaporator. Finally, from the 1 g of rowanberries, 1 mL of each aqueous extract variant was prepared.

### 4.5. Determination of Total Phenolic Content

The total phenolic content (TPC) was determined using the modified Folin–Ciocâlteu method [58], as follows: 0.1 mL of gallic acid solutions with concentrations of 0.01, 0.05, 0.10, 0.15, 0.20, and 0.25 mg/mL, respectively, were mixed with 0.25 mL of Folin–Ciocâlteu’s reagent, and after 3 min, 3 mL of 10% calcium carbonate solution was added. After 40 min in the darkness, the absorbances of the prepared calibrating solutions were measured at 765 nm using a UV–Vis Varian Cary 300 spectrophotometer (Thermo Fisher Scientific, Waltham, MA, USA). Then, the absorbance of appropriately diluted methanolic and aqueous extracts of rowanberries prepared analogously to the standard samples was measured similarly. TPC was determined based on the obtained calibration curve (y = 4.018x, R^2^ = 0.9999), and the results were expressed as mg of gallic acid per 1 g of extract dw.

### 4.6. Study on the Effect of Sorbus aucuparia Fruit Extracts on Fusarium Growth

The antifungal effect of rowanberries was estimated using a modified method of Uwineza et al. (2022) [56]: 15 mL of a mixture of PDA medium with 10% aqueous rowanberry extract was inoculated with a 5 mm mycelium of *F. proliferatum* (PEA 1) or *F. culmorum* (KF 846) on each Petri dish. The control group was PDA medium containing the investigated fungal strains without extracts. All samples were incubated at 25 °C in the darkness for 10 days in three replications. After 10 days of the experiment, the radial mycelial growth was measured, and the antifungal properties of each extract variant were calculated based on the following formula:

(2) $$mycelium\;growth\;inhibition=\frac{D_{c-}D_{e}}{D_{c}}\times 100\%$$

where *D_c_* is the average diameter of the control group (PDA medium with *F. proliferatum* or *F. culmorum*), and *D_e_* is the average diameter of tested samples (*Fusarium* spp. with PDA medium treated by rowanberry extracts).

### 4.7. Determination of Ergosterol Content

To 0.1 g of dried mycelium samples, 2 mL of methanol and 0.5 mL of 2 M sodium hydroxide were added. Samples were microwave-heated 3 times at 370 W for 20 s. After cooling, the samples were neutralized with 1 mL of 1 M hydrochloric acid and 2 mL of methanol. The suspensions were extracted three times with 4 mL of n-pentane, and then the solvent was air-evaporated. The dried residues were dissolved in 1 mL of methanol, filtered through 15 mm syringe filters with a 0.20 μm pore diameter (Chromafil, Macherey-Nagel, Duren, Germany), and analyzed by the UPLC-PDA technique according to Uwineza et al. (2022) [56]. The ERG content was given in µg per g of sample, and the ERG reduction percentage was calculated by the given formula:

(3) $$ERG\;reduction=\frac{E_{c-}E_{e}}{E_{c}}\times 100\%$$

where *E_c_* is the ergosterol content in the control group (PDA medium with *F. proliferatum* or *F. culmorum*), and *E_e_* is the ergosterol content in the test samples (PDA medium with *Fusarium* spp. treated with rowanberry extracts).

### 4.8. Analysis of Mycotoxins

Mycotoxins were extracted from the dried mycelium samples (0.335–0.506 g) by adding 7 mL of methanol and stirring for 24 h. Then, the samples were centrifuged at 5000 rpm for 10 min, and the supernatants were filtered using a disc filter with a 0.20 μm pore diameter (Chromafil, Macherey-Nagel, Duren, Germany). Mycotoxin detection and determination were performed with UHPLCHESI-MS/MS using a non-porous C18 Cortecs chromatographic column (100 mm × 2.1 mm × 1.6 μm, Waters, Manchester, MA, USA). The mobile phase consisted of water–methanol 90:10 (A) and methanol–water 90:10 (B); both phases had 5 mM ammonium formate and 0.2% formic acid. The separation parameters and operation of the chromatograph are given in detail in a previous publication [56]. The percentage of mycotoxin biosynthesis reduction by *F. proliferatum* and *F. culmorum* was determined using the following formula:

(4) $$mycotoxin\;reduction=\frac{M_{c-}M_{e}}{M_{c}}\times 100\%$$

where *M_c_* is the mycotoxin content in the control group (PDA medium with *F. proliferatum* or *F. culmorum*), and *M_e_* is the mycotoxin content in the tested samples (PDA medium with *Fusarium* spp. treated with rowanberry extracts).

### 4.9. Statistical Analysis

Experimental data were statistically evaluated using the Statistica 14 software package (TIBCO Software Inc., Palo Alto, CA, USA). A one-way analysis of variance (ANOVA) was used to calculate the means and standard deviations and to assess the significance of the differences in the effects of individual extracts. Subsequently, the post hoc Tukey’s honest significant difference (HSD) test was used for paired comparisons (*p* = 0.05).

## 5. Conclusions

This study aimed to determine the influence of *Sorbus aucuparia* fruit extracts obtained by a supercritical fluid extraction method under different conditions on *Fusarium proliferatum* and *F. culmorum* growth and mycotoxin biosynthesis. It was observed that the obtained extracts inhibited the growth of only *F. proliferatum*. However, the decrease in ergosterol concentrations showed that the growth of both *Fusarium* species was reduced, but this effect was more evident for *F. proliferatum* than *F. culmorum*. Rowanberry extract obtained at 70 °C and 300 bars showed the most significant antifungal activity, with an ERG reduction of 60.67% for *F. proliferatum* and 41.62% for *F. culmorum*. Despite a significant decrease in ERG content in both *Fusarium* spp., which indicated fungi reduction, there was a diversification in their biosynthesis of secondary metabolites. All tested extract variants reduced the production of BEA by *F. proliferatum*. However, fumonisins showed substantial increases in their biosynthesis compared to the control group. This phenomenon can be attributed to the fungal survival mechanism that stimulates mycotoxin production under specific stress conditions. Instead, extracts obtained from rowan fruits consistently decreased the production of all tested mycotoxins by *F. culmorum*. All prepared extract variants similarly reduced the content of DON, ZEN, and ZEN-14S. However, the most significant reduction in 3- and 15-AcDON content occurred when samples were treated with the extract prepared at 40 °C and 200 bar, and in the case of α-ZOL, with the extract prepared at 70 °C and 200 bar. The obtained results are important for practically assessing the effectiveness of *Sorbus aucuparia* fruit extracts against *Fusarium* pathogens in cereal crops. Future studies on the gene expression of the tested *Fusarium* spp. are planned to understand the mechanisms of their growth inhibition and the different mycotoxin biosynthesis effects. The current research shows great potential for advancing sustainable agricultural practices, addressing the challenges of fungal diseases in cereal crops, and enhancing food safety.

## Figures and Tables

**Table 1:** List of the prepared extract variants (E1–E4), including extraction conditions and extraction yield.

Extract Variant	Extraction Conditions	Extraction Yield [%]
E1	40 °C, 300 bar	17.71 ± 0.10 ^b^
E2	40 °C, 200 bar	16.93 ± 0.10 ^c^
E3	70 °C, 300 bar	18.05 ± 0.06 ^a^
E4	70 °C, 200 bar	16.99 ± 0.08 ^c^

All values are means of three replicates ± standard deviation. The values assigned with the superscripts of different letters are significantly different (Tukey’s HSD test, significant at *p* < 0.05).

**Table 2:** The percentage of ergosterol (ERG) reduction for *Fusarium* spp. samples treated with different variants of rowanberry extracts (E1–E4).

Extract Variant	ERG Content Reduction [%]	
	*Fusarium proliferatum*	*Fusarium culmorum*
E1	12.41 ± 14.07 ^c^	8.01 ± 11.41 ^b^
E2	27.30 ± 10.29 ^bc^	7.70 ± 5.62 ^b^
E3	60.67 ± 5.89 ^a^	41.62 ± 11.48 ^a^
E4	37.85 ± 1.92 ^ab^	22.42 ± 4.03 ^ab^

All values are means of three replicates ± standard deviation. The values assigned with the superscripts of different letters are significantly different (Tukey’s HSD test, significant at *p* < 0.05).

**Table 3:** The percentage of mycotoxin increase or decrease in tested samples of *F. proliferatum* treated with different variants of rowanberry extracts (E1–E4).

Extract Variant	Variation of Mycotoxin Content [%]			
	Increase			Decrease
	FB_1_	FB_2_	FB_3_	BEA
E1	242.9 ± 41.4 ^a^	180.5 ± 72.4 ^a^	216.3 ± 93.8 ^a^	82.5 ± 11.3 ^a^
E2	354.2 ± 54.4 ^a^	208.5 ± 61.3 ^a^	286.5 ± 105.5 ^a^	90.4 ± 4.9 ^a^
E3	1017.9 ± 481.1 ^b^	123.1 ± 65.6 ^a^	716.1 ± 216.1 ^b^	92.1 ± 5.6 ^a^
E4	704.1 ± 75.6 ^ab^	126.2 ± 63.0 ^a^	344.5 ± 142.1 ^ab^	80.6 ± 8.8 ^a^

FB_1_—fumonisin B_1_, FB_2_—fumonisin B_2_, FB_3_—fumonisin B_3_, BEA—beauvericin. All values are means of three replicates ± standard deviation. The values assigned with the superscripts of different letters are significantly different (Tukey’s HSD test, significant at *p* < 0.05).

**Table 4:** The percentage of mycotoxin reduction in tested samples of *F. culmorum* treated with different variants of rowanberry extracts (E1–E4).

Extract Variant	Mycotoxin Content Reduction [%]				
	DON	3- and 15- AcDON	ZEN	ZEN-14S	α-ZOL
E1	66.1 ± 3.1 ^b^	33.8 ± 10.3 ^b^	83.2 ± 1.2 ^a^	82.5 ± 3.2 ^a^	79.0 ± 18.9 ^ab^
E2	86.8 ± 6.1 ^a^	60.0 ± 12.6 ^ab^	89.1 ± 10.1 ^a^	82.3 ± 12.1 ^a^	89.2 ± 18.7 ^a^
E3	86.6 ± 2.3 ^a^	59.0 ± 11.6 ^ab^	43.6 ± 12.4 ^b^	79.8 ± 2.3 ^a^	32.7 ± 9.3 ^b^
E4	95.1 ± 2.3 ^a^	85.5 ± 9.2 ^a^	74.7 ± 12.8 ^a^	78.1 ± 4.1 ^a^	69.2 ± 26.6 ^ab^

DON—deoxynivalenol, 3- and 15-AcDON—3- and 15-acetyldeoxynivalenol, ZEN—zearalenone, ZEN14S—zearalenone-14-sulfate, α-ZOL—α-zearalenol. All values are means of three replicates ± standard deviation. The values assigned with the superscripts of different letters are significantly different (Tukey’s HSD test, significant at *p* < 0.05).

## Data Availability

Dataset available on request from the authors.
